# Eating Disorders in Sexual and Gender Minority Adolescents

**DOI:** 10.1007/s11920-024-01508-1

**Published:** 2024-06-03

**Authors:** Jason M. Nagata, Elena Stuart, Jacqueline O. Hur, Smriti Panchal, Patrick Low, Anita V. Chaphekar, Kyle T. Ganson, Jason M. Lavender

**Affiliations:** 1 Division of Adolescent and Young Adult Medicine, Department of Pediatrics, University of California, San Francisco, 550 16th Street, 4th Floor, Box 0503, San Francisco, 94143 CA USA; 2 Factor-Inwentash Faculty of Social Work, University of Toronto, Toronto, ON Canada; 3 Military Cardiovascular Outcomes Research Program (MiCOR), Department of Medicine, Uniformed Services University, Bethesda, MD USA; 4 Department of Medicine, Uniformed Services University, Bethesda, MD USA; 5 The Metis Foundation, San Antonio, TX USA

**Keywords:** Eating disorders, LGBT, LGBTQ+, Transgender, Gay, Lesbian, Bisexual

## Abstract

**Purpose of Review:**

To consolidate recent literature addressing eating disorders and disordered eating behaviors among sexual and gender minority (SGM) adolescents, including but not limited to lesbian, gay, bisexual, transgender, and queer (LGBTQ) adolescents.

**Recent Findings:**

Sexual and gender minority adolescents are at heightened vulnerability to eating disorders and disordered eating behaviors compared to their cisgender and heterosexual peers, potentially due to minority stress, gender norms, objectification, and the influence of the media, peers, and parents. We report findings from recent literature on the epidemiology and prevalence, assessment, mental health comorbidity, quality of life and psychosocial functioning, risk and protective factors, and treatment and interventions for eating disorders in sexual and gender minority adolescents.

**Summary:**

Addressing eating disorders in sexual and gender minority adolescents requires an integrated approach consisting of screening, tailored treatment, and comprehensive support to address intersectional challenges. Gender-affirming and trauma-informed care approaches may be considered.

## Introduction

Millions of adolescents worldwide suffer from eating disorders, which are serious mental health conditions characterized by persistent disturbances in eating or related weight control behaviors that significantly impair health and/or functioning [1, 2]. Eating disorders defined in the DSM-5 include anorexia nervosa, bulimia nervosa, binge-eating disorder, and avoidant/restrictive food intake disorder (ARFID), which are defined by varying physical (e.g., low body weight or significant weight loss, nutritional deficiencies), psychological (e.g., fear of weight gain, overvaluation of body weight/shape), and behavioral (e.g., binge eating, purging, excessive exercise) symptoms [2]. Many individuals also may exhibit patterns of symptoms that do not fully meet criteria for these core diagnoses but are associated with significant distress and impairment. Such cases are characterized as other specified feeding or eating disorders (OSFED), examples of which include atypical anorexia nervosa, purging disorder, and low frequency/limited duration forms of bulimia nervosa or binge-eating disorder [2]. Importantly, disordered eating behaviors (e.g., loss-of-control eating, unhealthy weight control strategies) can occur independently of a full syndrome eating disorder, and may be associated with greater risk for development of a full-threshold eating disorder over time [3].

A recent review reported that worldwide estimates of the lifetime prevalence of any eating disorder among male- and female-identified adolescents and young adults range between 0.6 and 26.7% [1]. Eating disorder prevalence estimates among U.S. adolescents specifically include 3.4–3.8% among female-identified adolescents, 1.2–1.5% among male-identified adolescents, and 2.7% for adolescents overall [1, 2, 4, 5]. Moreover, a systematic review and meta-analysis of studies that examined disordered eating using the SCOFF (a widely used 5-item eating disorder screening tool which is an acronym for Sick, Control, One, Fat, Food) [6] reported that, based on 32 studies from 16 countries, a little more than one in five adolescents exhibited disordered eating [7]. Notably, adolescence into young adulthood has been identified as a period of peak risk for eating disorder onset [5, 8], highlighting the importance of understanding eating disorders and disordered eating behaviors in adolescents, particularly among adolescent populations that may be at heightened risk.

An ever growing body of literature suggests that sexual and gender minorities (SGM) exhibit elevated vulnerability for body dissatisfaction, disordered eating behaviors, and eating disorders, likely due to an interplay between stigma and societal bias, minority stress, and other social pressures [9–11]. Sexual and gender minority populations include individuals who identify as lesbian, gay, bisexual, queer, or questioning (sexual minority), as well as those who identify as transgender, gender-diverse, gender-fluid, or gender-expansive (gender minority). Although much of the literature in this area has focused on adults, adolescence is a developmental period of significant physical, emotional, and social change that presents increased complexity for sexual and gender minority adolescents facing identity formation and societal stigma [12]. Consistent with this notion, sexual and gender minority adolescents may be particularly vulnerable to body image concerns and eating disorder symptomatology [13••, 14–17, 18•, 19]. For example, about 54% of sexual and gender minority adolescents receive an eating disorder diagnosis during their lifetime, with an additional 21% suspecting they have had an eating disorder or experienced disordered eating behaviors during their lifetime. Sexual and gender minority adolescents who received an eating disorder diagnosis (versus those with no suspected or diagnosed eating disorder) also have nearly fourfold greater odds of a past-year suicide attempt [20], further highlighting the importance of eating disorder screening, detection, and treatment in this population.

The literature on eating disorders and disordered eating behaviors in sexual and gender minority adolescents continues to grow quickly, providing new data to better inform our understanding of the nature, prevalence, risk factors, interventions, and other considerations related to eating disorder symptomatology in this vulnerable population. As such, this narrative review focuses on the most recent literature (past 3–5 years) addressing eating disorders and disordered eating behaviors among sexual and gender minority adolescents. We first review general theoretical frameworks to provide context for understanding eating disorders in sexual and gender minority adolescents, and then offer a review of recent findings related to epidemiology, assessment, mental health comorbidities, quality of life, risk/protective factors, and interventions. We conclude with a discussion of clinical implications, limitations, and future directions for the research in this area.

## Theoretical Framework

Several theoretical models addressing the onset, maintenance, and experience of mental and behavioral health concerns, including body dissatisfaction and eating disorders, are of strong relevance to understanding eating disorder symptomatology among sexual and gender minority adolescents. Perhaps of most relevance is Minority Stress Theory, which posits that sociopolitical forces promote social and structural contexts that disproportionately expose sexual and gender minority individuals to distal/external (e.g., discrimination, harassment, violence) and proximal/internal (e.g., internalized homophobia or transphobia, minority identity concealment) stressors [21, 22, 23••]. For example, in a nationally representative sample of 7,000 LGBT middle and high school students, the Gay Lesbian and Straight Education Network (GLSEN) found that participants reported extensive homophobic and transphobic bullying: 67.4% faced homophobic remarks, 85.2% experienced verbal harassment, and 95.7% faced negative remarks concerning gender nonconformity [24, 25•]. These identity-based stressors are conceptualized as being an important mechanism underlying observed mental and behavioral health disparities among sexual and gender minority individuals, including in relation to body image concerns and eating disorder symptoms [23••, 25•, 26].

Other models that focus on external pressures and internal experiences related to body weight/shape are also relevant to conceptualizing vulnerability to eating disorder symptomatology in sexual and gender minority adolescents. For instance, the Tripartite Influence Model highlights the salience of influences from peers, parents, and media. These pressures are posited to promote appearance comparisons and internalization of sociocultural appearance ideals, which in turn heighten risk for body dissatisfaction and eating disorder symptoms [27]. Similarly, the Dual-Pathway Model posits that social pressures to conform to body ideals and internalization of those ideals lead to body image concerns that, in turn, promote dieting behavior and negative affective experiences that increase eating disorder vulnerability [28]. Objectification Theory further contributes to these perspectives by illustrating how societal and cultural objectification fosters the internalization of self-objectification that exacerbates body surveillance and other tendencies that lead to adverse mental and behavioral health outcomes [29]. Although originally proposed as a framework specifically for understanding the experience of body image among women, concepts within the model have since been applied more broadly, including among sexual and gender minority individuals [30–32].

**Fig. 1:**
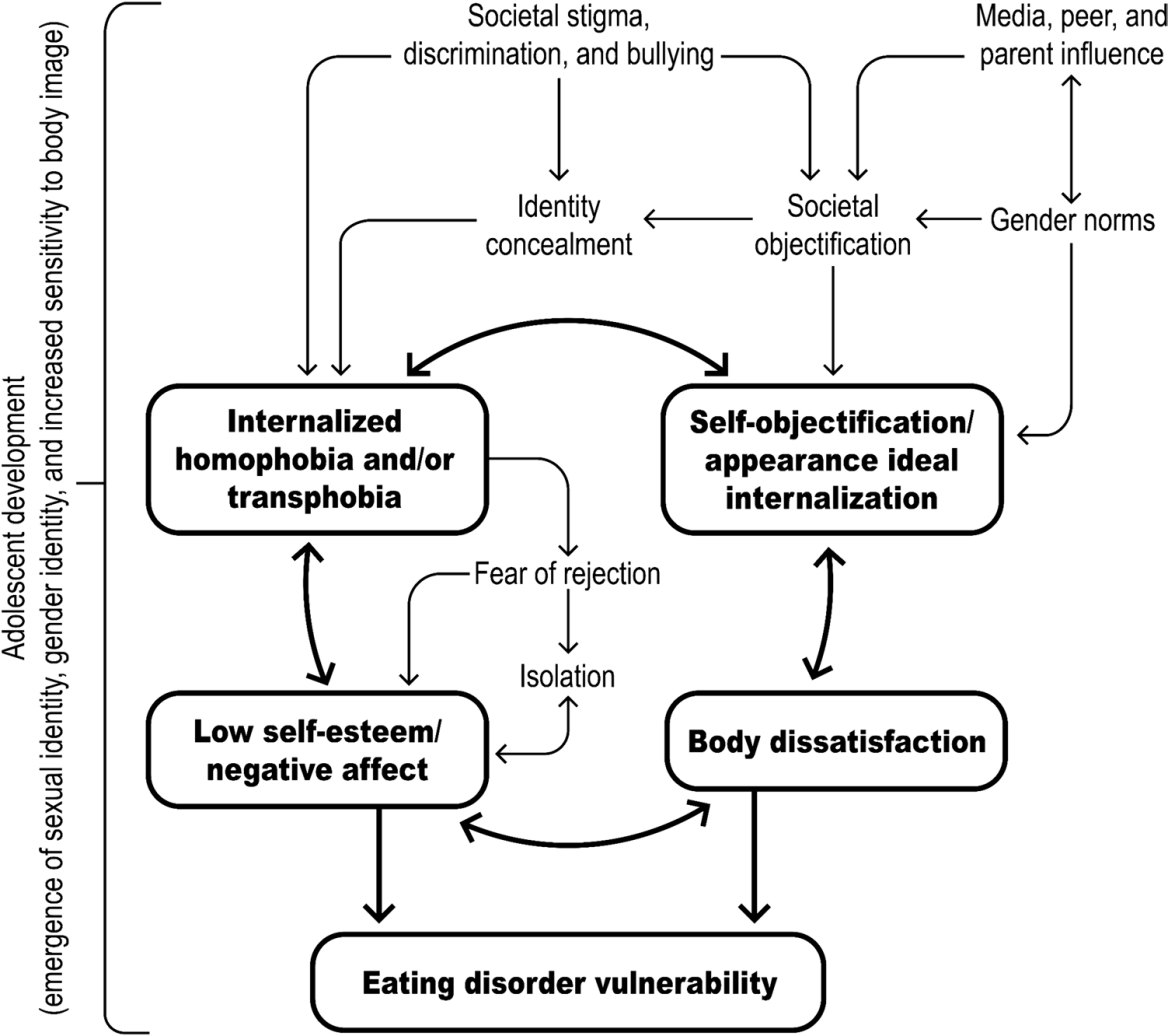
Integrated conceptual framework for understanding eating disorder vulnerability in sexual and gender minority adolescents

More recently, researchers have synthesized various theoretical models to investigate vulnerability to body dissatisfaction and symptoms of eating disorders among sexual and gender minority individuals. For example, the sources of social pressure identified in the Tripartite Influence Model may amplify body dissatisfaction and increase eating disorder symptom risk through the promotion of sociocultural appearance standards and appearance comparison processes, which may exacerbate the impact of stigma and stressors identified in the Minority Stress Model [22, 33]. Moreover, heightened self-surveillance as described in Objectification Theory may align with and intensify the adverse consequences of pressures and stressors delineated within both the Minority Stress and Tripartite Influence Models, potentially heightening body image concerns and vulnerability to eating disorder symptoms among individuals striving to conform to societal and gender norms [34]. Together, these frameworks elucidate a vicious cycle where societal discrimination, internalized stigma, and external pressures converge, magnifying the susceptibility of sexual and gender minority adolescents to eating disorders [35]. Moreover, for sexual and gender minority adolescents, the converging and interacting impacts of these external pressures/stressors and internal experiences/processes in relation to eating disorder symptom vulnerability occur within the broader context of adolescent development, which is characterized by the emergence of sexual and gender identity, increasing importance of peer influences, and other socioemotional and physical changes. Figure 1 presents a conceptual framework for understanding eating disorder vulnerability among sexual and gender minority adolescents.

## Review of Recent Literature

### Epidemiology and Prevalence

There is a considerable prevalence of disordered eating behavior among sexual and gender minority adolescents. In a recent cross-sectional survey of 8,814 sexual and gender minority adolescents aged 13–17 years old from the United States, 16.6% of participants were found to have at least one disordered eating behavior [36•]. Binge eating (9.7%) represented the most common behavior, followed by caloric restriction (6.2%), purging (3.2%), diet pill use (1.4%), and less commonly laxative use (0.8%) [36•].

Additionally, sexual minority (SM) identification may increase the risk of disordered eating behavior. Among a nationally representative sample of American adolescents, sexual minority identification was associated with a two-fold increase in odds of binge-eating disorder compared to heterosexual peers [14]. Similarly, Parmar et al. found the odds of disordered eating behavior in sexual minority adolescents to be two times higher compared to non-SM adolescents, along with an increased likelihood of body dissatisfaction [18•]. Risk of disordered eating behavior is further stratified among sexual minority subgroups including gay men and bisexual women who report higher rates of anorexia nervosa and bulimia nervosa relative to their heterosexual counterparts and other sexual and gender minority subgroups [15–17]. Sexual minority adolescents and young adults also experience greater symptoms of muscle dysmorphia compared to their heterosexual peers [37].

Compared to sexual minority adolescents, gender minority adolescents experience eating disorders at an even greater prevalence and severity [1, 7]. Roberts et al. found that transmasculine adolescents engage in multiple disordered eating behaviors including binge eating (15.1%), purging (7.0%), and diet pill use (3.0%) while non-binary birth-assigned female adolescents commonly engaged in caloric restriction (9.5%) [36•]. In a longitudinal study involving 91 gender minority adolescent participants, a significant proportion (40%) reported at least one disordered eating behavior, with 17% reporting at least three. The most common behaviors included exclusion of certain foods (30%), restriction of food quantity (27%), and binge eating (26%) [38•]. There is currently a scarcity in comprehensive longitudinal research on disordered eating behaviors in sexual and gender minority adolescents which we speculate may be caused by underreporting due to stigma and healthcare access barriers limiting adequate sampling. Future studies should attempt to further elucidate the prevalence of disordered eating behaviors in sexual and gender minority adolescents by sampling from diverse sexual and gender minority subgroups.

### Assessment

The most widely used eating disorder measures were typically developed based on traditional conceptualizations of eating disorder symptomatology that centered on the experiences of predominantly white and socioeconomically advantaged young women and have focused nearly exclusively on the thin body ideal and drive for thinness. More recently, research has begun to address the applicability, utility, and limitations of existing measures for use among more diverse populations, including sexual and gender minority individuals. Specifically, topics of consideration have included the extent to which existing measures accurately and comprehensively capture the potentially unique experience and manifestation of eating disorder symptoms in sexual and gender minority populations, and the need for culturally sensitive questionnaires, screening tools, and diagnostic measures that incorporate non-stigmatizing language and mirror the diverse experiences of sexual and gender minority individuals [39–41].

The Eating Disorder Examination-Questionnaire (EDE-Q) is one of the most commonly used measures in the eating disorder field, and has been used in numerous studies with sexual and gender minority samples [34]. EDE-Q norms have been published for a variety of adult sexual and gender minority populations [40, 42–46]. However, numerous studies of adult sexual and gender minority individuals have failed to replicate the original four factors of the EDE-Q (i.e., restraint, eating concern, shape concern, weight concern) [47, 48]. Although there have been fewer studies, recent findings in sexual and gender minority adolescents have been similar. For example, in a sample of gender-affirming treatment-seeking transgender adolescents and young adults ages 11 to 24, Peterson and colleagues (2020) [49] reported a unidimensional structure for the EDE-Q based on exploratory factor analysis. The authors recommended use of a total score for the EDE-Q, rather than use of the traditional subscales. Similarly, a recent study examining the psychometric properties of a 12-item short-form version of the EDE-Q among transgender and gender diverse young adults found good support for reliability and validity of the total score [50]. Moreover, other research using the EDE-Q with transgender adolescents found that many participants endorsed weight-change behaviors for gender-affirming purposes, which were generally discordant with EDE-Q scores, but could increase risk for development of an eating disorder over time [51].

Additional recent research has sought to validate other eating disorder measures in sexual and gender minority populations. For example, Zickgraf and colleagues [52] found good support for the Nine-Item Avoidant/Restrictive Food Intake Disorder Screen in a sample of transgender and nonbinary adolescents and young adults. Linsenmeyer and colleagues [53] also recently reported positive findings regarding the psychometric properties of the SCOFF (Sick, Control, One Stone, Fat, Food) screening questionnaire in a sample of transgender adolescents. However, despite progress in the assessment of eating disorder symptoms among sexual and gender minority groups, most of the existing research has focused on samples of adults, and more research on assessment among sexual and gender minority adolescents specifically is needed. Moreover, there remains a need to better understand and implement culturally sensitive, gender-affirmative approaches to eating disorder assessment in research and clinical settings [40, 41, 54].

### Mental Health Comorbidity

Co-occurring mental health concerns are common among those with eating disorders, and recent findings suggest that, compared to their cisgender and heterosexual peers, sexual and gender minority individuals with eating disorders in particular demonstrate higher rates of mental health comorbidities [14, 16, 41, 55••]. For example, in a recent retrospective chart review study of patients admitted to a large inpatient eating disorder medical stabilization unit, Chaphekar and colleagues [55••] found that more than one in five identified as a sexual minority. Adolescent and young adult sexual minority patients had higher rates of co-occurring depression, anxiety, and post-traumatic stress disorder, and were also more likely to have a history of self-injury or suicidality. Similarly, in a recent nationwide study of U.S. college students (approximately 19 years of age on average), participants with a probable eating disorder who identified as bisexual (vs. heterosexual) were more likely to screen positive for possible depression, and those who identified as other sexual orientation (e.g., queer, questioning, other, or multiple sexual orientations vs. heterosexual) were more likely to screen positive for a probable anxiety disorder [56]. Moreover, gender minority students with a probable eating disorder were more likely than cisgender males to screen positive for probable depression or anxiety disorder.

In another recent investigation, Kasson and colleagues [57] recruited adolescents who screened positive for a clinical/sub-clinical eating disorder or were at risk for an eating disorder for a study examining an eating disorder digital intervention. Of the 134 enrolled participants, the majority (70%) identified as a sexual or gender minority. A large majority of participants scored in the moderate or higher range on measures of anxiety (87.3%) and depression (83.3%), and approximately one in three also endorsed a lifetime suicide attempt. Finally, recent findings from a large nationwide study of sexual and gender minority adolescents also found that approximately one in six met the clinical threshold for at least one disordered eating behavior, with binge eating behavior found to be the most common [36•]. Analyses examining psychological factors in relation to meeting clinical threshold of various disordered eating behaviors revealed that depressive symptoms were significant for all disordered eating behaviors, whereas self-esteem was associated with only certain disordered eating behaviors, and general stress was not significant for any disordered eating behaviors. Moreover, moderation analyses revealed that a number of associations were especially strong for gender minority versus sexual minority participants [36•].

Societal stigma and other identity-related stressors likely exacerbate both eating disorder symptoms and co-occurring mental health concerns in sexual and gender minority individuals, with structural barriers having further negative impacts on the mental health outcomes in these populations [39, 58]. The association between eating disorder symptoms and mental health comorbidities, particularly during the sensitive period of adolescent development and coupled with barriers to screening and treatment, highlights the nuanced challenges faced by sexual and gender minority adolescents and the importance of accessible, gender-affirming approaches to mental and behavioral healthcare [58]​​. Moreover, despite the pronounced severity of eating disorder symptoms and mental health comorbidities among sexual and gender minority individuals, research on this topic in samples of sexual and gender minority adolescents remains limited, and more investigations are needed.

### Quality of Life and Psychosocial Functioning

Quality of life is a broad construct that has been defined as “an individual’s perception of their position in life in the context of the culture and value systems in which they live and in relation to their goals, expectations, standards and concerns” [59]. Evidence indicates that reduced quality of life is common among individuals with eating disorders, at times surpassing impairments associated with other mental and physical health conditions, such as depression and anxiety disorders, substance use disorders, schizophrenia, diabetes, cardiovascular diseases, and chronic pain disorders [35, 60–62]. Among sexual and gender minority adolescents, the complex interplay between minority status and the psychosocial impacts of eating disorder symptoms may impede the formation of a positive self-image and social connections, which are cornerstones of healthy adolescent development and are crucial for the transition into young/emerging adulthood.

In addition to higher rates of psychiatric comorbidity, self-injurious behaviors, and suicidality among sexual and gender minority adolescents compared to their cisgender and heterosexual peers, functioning in other domains may also be impacted [41, 55••, 63]. For instance, in a sample of college students, Simone and colleagues [17] found that sexual minority students were more likely to report eating pathology-specific academic impairment compared to their heterosexual peers. Moreover, a study of transgender adolescents found reduced health-related quality of life, particularly in psychological and physical domains, as well as social support, peer, and school-related areas of well-being [64].

Notably, research shows that perceptions of school safety are associated with fewer unhealthy weight control behaviors and less binge eating in sexual and gender minority adolescents, highlighting the importance of safe and supportive academic settings for students with diverse identities [65]. Additionally, the link between eating disorders and academic impairment in sexual minority college students underlines the potential impact of these conditions on educational outcomes [17], emphasizing the salience of inclusive educational practices and settings to support diverse identities. Education is a crucial determinant of future success and overall quality of life, making it imperative that sexual and gender minority adolescents have access to safe and equitable educational opportunities [66, 67].

### Risk and Protective Factors

Concealment pressures and minority stressors, such as low income, ethnic minority status, and non-conforming identities, contribute to increased eating disorder prevalence and severity among sexual and gender minority individuals [14, 16]. While societal stigma impacts all sexual and gender minorities, sexual minorities face orientation-linked challenges unique to their sexual minority subgroups. For example, adolescent lesbians are more likely to report higher BMIs while gay male adolescents are more likely to report being influenced by appearances portrayed in media [16]. Meanwhile, gender minorities experience identity-related issues including distress around undesired traits associated with their assigned sex [16]. Lack of self-acceptance and adherence to perceived societal appearance standards in these instances result in higher rates of disordered eating behaviors [36•].

Sexual and gender minority adolescents with positive feelings towards their identity have been shown to have reduced disordered eating behaviors, whereas factors such as stress associated with ‘coming out’, increase disordered eating behaviors [36•]. Additional protective factors such as safe school environments, social support, stable relationships, and self-compassion further mitigate minority stress effects and safeguard against eating disorder symptoms in sexual and gender minority adolescents [16, 65]. In their review, Espelage et al. find that school policies promoting education of sexual orientation, gender identity, and gender expression reduce homophobic language and verbal harassment [25•]. In schools that explicitly condemn homophobic bullying, respond swiftly to such incidents, and incorporate LGBT topics into their curriculum, sexual and gender minority students report increased feelings of belonging and reduced truancy rates [25•]. Although underexplored in the literature, familial compassion and understanding also contribute to decreased fear of victimization from parents based on sexual orientation [68]. Family acceptance also markedly diminishes the prevalence of suicidal thoughts and suicide attempts among sexual minority individuals with nearly half the individuals from highly accepting families reporting these symptoms compared to individuals with low family acceptance [69].

Pubertal changes, on the other hand, lead to increased gender incongruence and body dissatisfaction in gender minority adolescents. For example, one longitudinal analysis of disordered eating behaviors in adolescents found that increasing age was associated with increased EDE-Q scores [38•]. Despite this, gender-affirming care and hormone therapy may play a role in decreasing disordered eating behaviors and improving one’s self-image. The same analysis found that EDE-Q scores stabilized 12 months after initiation of gender-affirming care [38•], while another investigation reported significant decreases in body dissatisfaction and modest mental health improvements after a year of hormone therapy. These interventions improve mental health outcomes for transgender adolescents, including reductions in suicidal ideation, attempts, and non-suicidal self-injury [70].

### Treatment and Interventions

Presentation variations of eating disorders and increased risk of psychiatric comorbidity in sexual and gender minority adolescents present a need for targeted interventions. In an investigation by Chaphekar et al., the authors found that sexual minority-identified adolescents hospitalized for eating disorder complications present at higher weights but similar degrees of vital sign instability compared to their heterosexual peers [30]. Gender minority adolescents present with even higher weights and degrees of vital sign instability and are hospitalized at younger ages than their cisgender peers [71].

Despite a significant prevalence of eating disorders in sexual and gender minorities, there are currently no treatments specifically targeted toward sexual and gender minority adolescents. Baker et al. investigated the augmentation of the family-based treatment (FBT) model through the incorporation of a peer and family mentor (FBT+) and found that the FBT + model was effective in reducing eating disorder symptoms and achieving weight gain or recovery in gender minority adolescents [72••]. However, more research on the long-term outcomes of this intervention is likely needed.

Gender-affirming care is a commonly recommended intervention by professional organizations including the American Academy of Pediatrics and Pediatrics Endocrine Society [73, 74••, 75]. However, studies on its efficacy in reducing eating disorder symptoms have yielded mixed results. While one qualitative study suggests that gender-affirming care can alleviate symptoms in certain gender minority adolescents, disordered eating behaviors often persist particularly when there are other mental health comorbidities [38•, 74••, 75–77]. Beyond gender-affirming care, addressing gender dysphoria concurrently with eating disorder symptoms and incorporating trauma-informed, inclusive care through an interdisciplinary team versed in gender-affirming care principles may be effective treatment strategies [78].

### Implications and Applications

Data on the efficacy of nutritional status monitoring and weight restoration in sexual and gender minority adolescents with eating disorders is limited, with ongoing controversy on the use of binary sex growth charts for gender minority adolescents [71, 74••, 75, 78, 79]. In a review of ten hospitalized gender-expansive adolescents with eating disorders, the authors found no significant clinical differences in malnutrition severity or treatment goal weights between the Centers for Disease Control and Prevention (CDC) boys’ and girls’ growth charts [71]. However, differences may emerge at higher weight percentiles [80]. Current recommendations suggest use of binary growth charts and subsequent adoption of the chart corresponding to the individual’s affirmed gender if differences are minimal to promote inclusive care [71, 80]. However, more research is needed to determine best practices for use of growth charts within this population.

Sexual and gender minority individuals encounter a range of identity-based traumas that influence disordered eating behavior [36•], yet the support from current systemic healthcare structures is limited. Medical treatments, such as gender reconstructive surgery, fertility preservation, and continued health screening for sexual and gender minority individuals, are incredibly complex [9, 58, 81], which can potentially result in more stress and fear regarding receiving treatment. During these treatments, sexual and gender minority individuals have expressed perceiving a lack of understanding and compassion from healthcare providers [9, 68], primarily due to a lack of education and perpetuation of cis-heteronormative treatment models. The confluence of identity-based trauma, societal pressures, and specific medical challenges faced by sexual and gender minority adolescents underscores the need to develop a knowledgeable and empathetic healthcare workforce capable of navigating sexual and gender minority individuals’ specific challenges and enhancing their health outcomes [9, 68, 81].

Policy reform within social and healthcare institutions may improve access to and outcomes of eating disorder treatment for sexual and gender minority adolescents [36•, 58]. Gender-affirming and trauma-informed interventions have yielded positive health outcomes, such as less emotional internalization, better psychological functioning, and a greater sense of belonging in sexual and gender minority adolescents with eating disorders [9, 68, 82]. Integration of sexual and gender minority health and eating disorder care into standard medical curricula may better enable healthcare providers to provide sensitive care and improve health outcomes in sexual and gender minority adolescents [9, 68, 83].

### Future Directions

Our review describes significant gaps in the research on eating disorders and disordered eating behaviors among sexual and gender minority adolescents. These areas focus primarily on diversifying study populations, tracking the prevalence of eating disorders and disordered eating behaviors among sexual and gender minorities over time, improving assessment tools, and developing inclusive treatment protocols.

There is a need to diversify study populations to ensure that the experiences of a wide breadth of sexual and gender minority adolescents are being documented. This will ensure that future research, theory, assessment, and treatment are tailored to the needs of the population. Tracking the prevalence of eating disorders and disordered eating behaviors among sexual and gender minority adolescents over time is critical to understanding how these disorders and behaviors evolve as one goes through crucial developmental milestones. Tracking trends over time will also provide valuable insights into additional risk and protective factors (e.g., relationships, careers, hormone stabilization post-puberty) that arise as one progresses through life. Additionally, tracking trends over time will offer insights into treatment outcomes and inform care optimization [74••], including identifying how gender-affirming treatment may be a critical intervention.

Assessments of eating disorders and disordered eating behaviors that are specific to the sexual and gender minority experience need to be developed. These should be validated among a diverse group of adolescents to ensure sensitivity and specificity. The inclusion of gender incongruence and minority stress experiences into assessment tools may also be particularly relevant and can assist with conceptualizing treatment needs, including developing inclusive treatment protocols that consider recent insights into sex differences [16, 35, 55••, 84]. Improving care for this vulnerable group requires addressing these existing gaps in our understanding.

Further exploration of the implications of hormone treatment or pubertal suppression on eating disorders within transgender adolescents is another crucial research avenue [85]. Recent advancements in understanding sex differences in eating disorder treatment inform personalized care [55••, 81, 83, 86], and future research should continue prioritizing compassionate and inclusive interventions attuned to sex differences and gender diversity. Indeed, highlighting the need for providers to be knowledgeable, empathetic, and inclusive is key to improving sexual and gender minority healthcare. Involving sexual and gender minority adolescents in the development and refinement of interventions to their specific experiences and needs and assessing how these efforts reduce societal stigma and minority stress may be one route to enhancing treatment outcomes for this group. This endeavor will require collaborative efforts across healthcare disciplines, integrating clinical insights with empirical research to pave the way for a more inclusive and effective healthcare paradigm for sexual and gender minority adolescents.

## Conclusions

Current research demonstrates challenges faced by sexual and gender minority adolescents in accessing and successfully receiving treatment for eating disorders. Despite the high prevalence of disordered eating behaviors in this population, there is a lack of targeted treatment options directed toward sexual and gender minority adolescents. Policy changes within social and healthcare institutions promoting inclusive care may improve access to and outcomes of eating disorder treatment for sexual and gender minorities. Future investigations recruiting from diverse sexual and gender minority populations should focus on the development of validated screening tools, adaptations, and recommendations for further institutional reform.

## Data Availability

No datasets were generated or analysed during the current study.

## Declarations

### Disclaimer

The opinions and assertions expressed herein are those of the author(s) and do not reflect the official policy or position of the Uniformed Services University or the Department of Defense.
